# Dr. Eugene B. Nash

**Published:** 1918-02

**Authors:** 


**Dr. Eugene B. Nash,** Cortland, N. Y., died Nov. 6, age 79. He was graduated from the Homeopathic College, Cleveland in 1874. He was formerly professor of materia medica in the New York Homeopathic Medical College.

